# Predator-Prey Relationship between Urban Bats and Insects Impacted by Both Artificial Light at Night and Spatial Clutter

**DOI:** 10.3390/biology11060829

**Published:** 2022-05-27

**Authors:** Han Li, Kenneth T. Wilkins

**Affiliations:** 1 Department of Biology, University of North Carolina Greensboro, Greensboro, NC 27412, USA; 2 Department of Biology, Baylor University, Waco, TX 76798, USA; ken_wilkins@baylor.edu

**Keywords:** bats, foraging activity, ALAN, spatial complexity, urban, insects, predator-prey relationships, trophic dynamics, artificial light at night

## Abstract

**Simple Summary:**

Artificial light at night provides convenience and a perception of safety for humans. However, it is an environmental pollutant to many wildlife species. We found that in the city artificial light at night attracted insects, which could be a source of food for bats. However, whether bats could use this food source depended on their ability to navigate through objects in space. We found that bats that preferred open habitats did not fully use insects attracted by artificial light at night when foraging sites were cluttered by fences, billboards, and other objects. In contrast, bats that were considered not to benefit from artificial light at night could take advantage of cluttered sites and be more active and forage more. Lastly, there were a few species absent in our study, which could be light-sensitive and not adaptive to urban environments. We suggest that limiting or reducing artificial light at night is needed to conserve bats.

**Abstract:**

Predators respond to the increase of prey by aggregation in space or foraging more often. However, foraging habitat suitability limits predators’ responses. For nocturnal insectivorous bats, artificial light at night (ALAN) can trigger insect prey aggregation. It is not clear how ALAN might affect predator-prey relationships in the urban setting, where urban bats could have adapted to the city, and novel spatial complexity introduced by man-made objects might alter foraging habitat suitability. We strategically selected sites to represent different levels of ALAN and spatial complexity. We recorded bat commuting and foraging activities and collected aerial insects to examine how ALAN and spatial complexity affected bat-insect relationships. We found that insect biomass was positively correlated with ALAN, but was not affected by spatial complexity. Large-sized big brown bats and hoary bats positively responded to change of prey in open sites whereas small-sized eastern red bats and silver-haired bats positively responded in cluttered sites, suggesting that the impact of ALAN could vary when ALAN is coupled with urban spatial complexity. Our study demonstrates that foraging habitat suitability can alter which species might benefit from ALAN. Predator-prey relationships in cities are complex, but general ecological principles still apply in novel urban ecosystems.

## 1. Introduction

Anthropogenic environmental changes have cascading effects on bats and their prey [1]. One important anthropogenic change that the world faces is ever-increasing artificial light at night (ALAN), which can have drastic negative consequences for many groups of animals [2,3,4,5]. A variety of insects is attracted by ALAN, many of which are the primary prey (i.e., Diptera, Lepidoptera, Coleoptera) of insectivorous bats [6,7,8,9,10]. Researchers also found that the defensive behaviors of some insects against predators can be compromised when flying under lights, making them more vulnerable to being captured by bats [11,12,13]. Thus, studies have documented that certain bat species tend to be more active and forage more in the presence of ALAN [14,15,16,17]. 

Field experiments have also demonstrated the negative impacts of ALAN on insectivorous bats. Several radio-telemetry or GPS tracking studies have shown avoidance of well-illuminated areas in bat flights [18,19,20,21]. When experimentally introducing ALAN to naturally unlit environments, ALAN disrupted flight patterns and reduced activity in many species, regardless of the light source types (light-emitting diodes/LED, sodium pressure, or mercury vapor) of ALAN [22,23,24,25,26]. Acoustic studies that focused on ALAN in urban settings also found negative impacts of ALAN on bat commuting activity [27,28,29], even though urban bats might have adapted behaviorally to the city [30,31,32]. 

A recent review of studies on ALAN and European bats concluded that species benefiting from ALAN are likely open or edge aerial foragers, preferring habitats with low spatial clutter. European species adapted to forest interiors of high clutter are more likely to be negatively impacted by ALAN [33]. Studies in South Africa [34], Panama [17], and Australia [18,35] also support this pattern. Very little is known about how North American bats respond to ALAN. A field experiment in Connecticut, USA, found that no bat species responded positively to ALAN when LED floodlights illuminated a dark nature preserve [24]. Similarly, another field experiment in the forest in Missouri, USA also found that most species avoided experimentally lit areas regardless of their foraging strategies except for the eastern red bat (*Lasiurus borealis*, species abbreviation LABO, [36,37]). So far, few studies have been conducted in urban areas to specifically study bats and ALAN in North America. 

Only a few studies on bats and ALAN conducted in Europe have incorporated aerial insect samples to examine the predator-prey relationship [26,38,39]. These studies found that ALAN altered insect communities and that more insects were captured when ALAN was introduced. They also demonstrated that bats utilizing the altered prey resource were open or edge aerial foragers whereas other species did not forage on the altered prey resource [26,38,39]. 

Many aspects of the bat-insect predator-prey relationship under ALAN deserve further investigation. Firstly, in urban environments where ALAN is most prevalent [2], manmade objects such as poles, billboards, fences, and even isolated buildings can increase spatial complexity and require additional effort for urban bats to navigate successfully. Those objects differ ecologically from the clutter of vegetation because trees provide a habitat for insect prey [40]. It is not known how increased urban spatial complexity might affect the bat-insect relationship under ALAN. Secondly, in urban environments, ALAN has different illumination levels for various human needs. It has been documented that bats’ responses to ALAN change with differences in light intensity [38,39]. Yet, it is not clear how different ALAN illumination levels in an urban setting might affect the bat-insect relationship. Thirdly, most published studies on bats and ALAN are not based on bat foraging calls, although they can be separated from regular commuting calls in acoustic recordings [41]. It is still not clear whether aggregation of insects due to ALAN might trigger aggregation of bats or whether bats might forage more often. 

To tackle the above questions concerning the dynamics of the bat-insect predator-prey relationship, we conducted a field experiment by strategically selecting sites to represent different levels of ALAN and spatial complexity. We sampled both bats and insects and further analyzed bat acoustic recordings to separate foraging calls. Our null hypothesis was that neither ALAN nor spatial complexity would affect insect availability, bat commuting activity, or bat foraging activity. We predicted that increased levels of ALAN would increase insect availability, indicated by higher total insect bat biomass and higher insect counts. We expected that spatial complexity would not have any effect on insect availability. We also predicted that bats would positively respond to increased insect availability, indicated both by more bat calls and by higher proportions of foraging calls being recorded at sites with higher levels of ALAN. Additionally, bats’ responses to ALAN would be affected by spatial complexity. For example, open foragers (such as the Mexican free-tailed bat, *Tadarida brasiliensis*, TABR, or the hoary bat, *Lasiurus cinereus*, LACI, [42]) would only respond to ALAN in open sites but not in cluttered sites, whereas opposite patterns might be found in edge or narrow space forager species. 

## 2. Materials and Methods

### 2.1. Study Area and Selection of Recording Sites

We set up the field experiment on the Baylor University campus in Waco, McLennan County, TX, USA. The campus has a typical low building density layout with many open spaces. Trees are scattered over the campus. The physical structure of buildings on the campus is relatively homogenous, but the lighting conditions vary in relation to function. For example, parking structures and sports fields are usually well-illuminated, whereas classroom buildings, dorms, and storage buildings have fewer or no exterior lights. Most lights are white or yellow. Some lights, such as decorative lights (mostly green), emergency call box lights (blue or red), and traffic lights (red and green) may display various colors. The type of lights also varies, including both traditional gas discharge lights and new LED lights. 

We selected a series of recording sites for bat detector deployment to encompass the range of intensities of ALAN. In a preliminary ground-truthing, we measured ALAN intensity throughout the campus using a light meter (EasyView 30, Extech Instruments Corp, Waltham, MA, USA) at multiple times of night and on nights representing different moon phases. Based on light intensity measurements and lighting regimes, we selected 12 sites to represent three ALAN levels (four sites per level): low illumination (within 50-m radius the mean of 20 random light intensity measurements was <25 lux,), medium illumination (within 50-m radius the mean of 20 random light intensity measurements was about 90 lux), and high illumination (within 50-m radius the mean of 20 random light intensity measurements was >250 lux). We made sure the half-sphere over the placement location of the bat detector had the same level of illumination. At all 12 sites, lights were illuminated the entire night and with the same lighting regime (lights on at least 15 min before sunset and off at least 15 min after sunrise) every night during the field data collection. 

Furthermore, within each ALAN level, we chose two sites to be open (no objects beside light poles within the 50-m radius, e.g., parking lots, open fields). The other two sites had objects that were at least 3 m tall and could clutter the space and increase spatial complexity. Objects included sports equipment, fences, billboards, scattered trees, sculptures, etc. (see Supplementary Material File S1 for photo illustrations). The objects that cluttered the space, however, did not form a structure to completely block bat flights or sound and light transmissions. We chose sites to have a similar density of clutter, about 5–10 objects within a 50-m radius. However, the types of objects varied among sites. 

All recording sites were located at least 400 m away from each other to ensure a bat would not be recorded simultaneously by two detectors. The farthest two recording sites were approximately 1.5 km apart to ensure that the same bat community was studied. Additionally, all sites were at least 50 m away from any natural water body and at least 200 m away from known bat roosts identified in a previous study [43]. We recognized that the responsiveness of insects to light varies with colors (wavelengths) of light ([9,44,45,46], but see different bat responses in [5,47,48,49]). However, usually, urban ALAN is a combined effect of many different types and colors of lights instead of a single source of homogenous light. Therefore, our sites represented a certain ALAN intensity level generated by multiple lights with different types of light bulbs in a more realistic manner. 

### 2.2. Acoustic Recording of Bats and Call Analysis

We used full-spectrum Song Meter SM2BAT bat detectors and SMX-II weatherproof acoustic microphones (Wildlife Acoustics, Inc., Maynard, MA, USA) to record bat acoustic activities. Between 22 September 2011, and 4 October 2012, we recorded bat activity continuously from sunset to sunrise every night, regardless of weather conditions. A total of four bat detectors were used to sample 12 sites with a three-week sampling cycle. During the first week of each cycle, we randomly chose one site from each of the three ALAN levels and a fourth random site to sample. In the second week, we randomly chose one not-yet-sampled site from each ALAN level; the fourth site was randomly chosen from the two ALAN levels not represented by the fourth site during the previous week. We sampled the remaining four sites during the third week. Thus, every three weeks a rotation of 12 sites would be completed and a new schedule would be generated. In this way, we minimized the temporal correlation of continuous recording among sites between weeks [50,51]. 

At all sites, the bat detector was installed on a metal pole on the ground or on building rooftops. We placed the microphone facing straight up, 1.8–2 m above the ground or rooftop. We made sure that there was no object (including a light) immediately adjacent to the microphone within 5 m. For sampling weather covariates, we extracted nightly minimum temperature from the National Oceanic and Atmospheric Administration weather database. All bat monitoring procedures were approved by Baylor University’s Institutional Animal Care and Use Committee (IACUC, protocol 200269-2).

Previous studies [43,52,53] conducted in the same city recorded big brown bat (*Eptesicus fuscus*, EPFU), eastern red bat (*Lasiurus borealis*, LABO), hoary bat (*Lasiurus cinereus*, LACI), silver-haired bat (*Lasionycteris noctivagans*, LANO), cave myotis (*Myotis velifer*, MYVE), evening bat (*Nycticeius humeralis*, NYHU), tricolored bat (*Perimyotis subflavus*, PESU), and Mexican free-tailed bat (*Tadarida brasiliensis*, TABR). Therefore, we only considered those species for bat acoustic analysis and species identification. Based on existing literature, we considered hoary and Mexican free-tailed bats open space foragers and other species edge foragers [42,54,55]. We defined a bat pass as a recording file of a call sequence including five or more echolocation calls separated by less than 1 s. Recordings with less than five echolocation calls were not analyzed. We used Kaleidoscope (version 4, Wildlife Acoustics, Inc., Maynard, MA, USA) to filter out recordings that met analysis criteria, then manually identified all bat passes to species using a call reference library (see [52,56] for how the reference library was developed). All manual identifications were conducted by the first author to maintain identification consistency. 

Based on manual identification results, we extracted species-specific bat activity (including all call types) as a dependent variable (named ‘bat activity’ in figures). To incorporate night length variation among months, we standardized species-specific bat activity as the number of passes per recording hour by dividing bat passes within a week over total hours of recordings [41]. We calculated species-specific bat activity using data collected in a week to avoid pseudo-replications among nights at the same sites [50]. We also calculated the weekly mean night temperature as a sampling covariate for weekly species-specific bat activity. 

As our goal was to analyze species-specific foraging activities, we further manually differentiated foraging calls with feeding buzzes (calls with increasing pulse repetition rates and cumulating in a rapid burst of calls as the bat closes on its target [41,57]) from regular commuting calls. We manually examined all bat passes that had been assigned to a species and searched for feeding buzzes. As we were interested in the species-specific response to ALAN, bat passes that could not be identified to species were not examined for foraging calls with feeding buzzes. If a bat pass included foraging calls with feeding buzzes, we defined it as a foraging pass. To represent how often bats foraged at a site, we defined a dependent variable as the foraging ratio by dividing the number of species-specific foraging passes over bat passes of the same species at a site for a sampling week [41,58]. 

### 2.3. Aerial Insect Sampling

We used unscented glue traps (approximately 50 cm by 40 cm, CatchMaster glue board trap, AP&G Co., Inc., New York, NY, USA) to capture nocturnal aerial insects. During each week, we chose one night (no rain, wind speed < 12 kph) to sample insects at all four sites with bat detectors. We tied the glue trap to a rectangular frame and clamped the frame 1.8 m above the ground to the poles supporting the bat detectors. In this way, the trap could not be moved by wind. The sampling period was from sunset to sunrise of the next day. We also recorded the minimum temperature for the corresponding night as the insect sample covariate. Although the insect samples might not have represented the particular insect assemblage that bats in this study were actually consuming, we considered them to serve as a reasonable proxy representing the greater insect community in the immediate vicinity. 

All glue traps were kept under a fume hood for 7 days to air-dry insects before identifying and measuring them on the trap. We measured the body length (from head to tip of abdomen) to 0.1 mm by using calipers under a dissecting microscope. We included only insects having body lengths 1–30 mm for further analyses, as bats might be less likely to consume prey larger or smaller [59]. We identified those insects with body lengths >3 mm to order (identification references [60,61]) and counted the number of insects per order per trap night. Insects 1–3 mm long were categorized as “small insects”. Using order-level identifications, we calculated the Shannon diversity index for each trap. 

We used the power model to estimate each insect’s weight by its length:

y = a(x)^b^ (1)

where: y = weight, x = length, and a and b are order-specific parameters described in existing literature [62,63,64,65]. For the small insect category, we used the general formula parameters for Class Insecta [62,63,64,65]. We added the weights of all aerial insects on the trap and recorded it as insect biomass (mg). We chose this method to estimate insect biomass instead of weighing each glue trap because the trap could capture dirt or organisms that could not be consumed by bats. 

### 2.4. Statistical Analyses

We conducted all statistical analyses and data visualization in R (version 4.1.0, R Development Core Team, Vienna, Austria [66]). In our experiment, there were two independent variables: ALAN level (low, medium, high) and spatial complexity (open vs. cluttered). We examined the interaction between the two variables. We tested temperature as a covariate and included it in statistical models whenever the temperature was found to affect the dependent variable. The other covariates considered included participation, wind speed, site distance to water, number of trees within a 50-m radius at the site, site ground cover type (concrete vs. soil), and site distance to interstate highway. None of these covariates were found significant and thus were not included in any analysis. We tested all dependent variables for normality by the Shapiro test. If a dependent variable was found not normally distributed, we chose other distributions accordingly [67]. We used 0.05 as the statistical significance criterion for all tests.

To evaluate the effects of ALAN and spatial complexity on bat activity, foraging ratio, and aerial insects, we constructed generalized linear models (GLM) for species-specific bat activity, species-specific foraging ratio, insect count, Shannon index for insects, or insect biomass, using ALAN level, spatial complexity, and their interaction term as independent variables. Low level for ALAN and cluttered for spatial complexity were used as the references for categorical variables in any GLM. If the interaction term was found not significant in any GLM, it was removed from the model. We modeled bat activity and insect biomass with the quasi-Poisson distribution, foraging ratio with the quasi-binomial distribution, insect count with the Poisson distribution, and Shannon index for insects with the gaussian distribution based on the preliminary analysis of dependent variable distributions. For any GLM with significant results involving ALAN, we conducted post hoc Tukey’s pairwise comparisons to compare levels of ALAN using R package “multcomp” [68]. 

## 3. Results

Between September 2011 and October 2012, we completed 17 sampling cycles (51 weeks in total) and obtained 353,638 acoustic recording files and 204 glue traps of insects. From 204 glue traps, we collected 18,822 insects that had a body length between 1 mm and 30 mm (insect count per trap 25% quantile 57.5, median 82, 75% quantile 122.5). We identified 5282 of them to 11 orders (Table S1). The most common insects were from Order Diptera with 3580 individuals (Table 1), followed by Coleoptera (634), Hymenoptera (379), and Homoptera (277).

**Figure 1:**
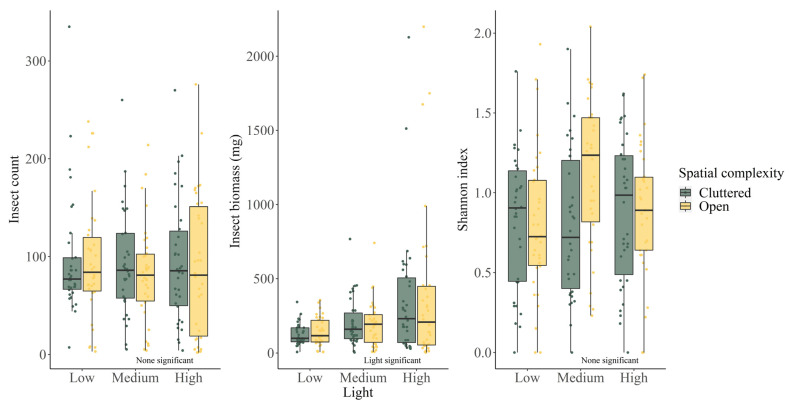
Boxplots (25th, 50th, 75th percentiles) and raw data points of insect count, Shannon diversity index, insect biomass at sites of varying ALAN levels, and spatial complexity. Independent variable significance is annotated in the graph.

Comparing across sites, we found no insect count or Shannon diversity index differences among all sites (all *p* > 0.05, Table 1). Higher insect biomass was found as site ALAN level increased (both *p* <0.05, Table 1, Figure 1). On average, insect biomass was 133.9 ± 85.0 mg/trap at low ALAN sites, 200.1 ± 156.1 mg/trap at medium ALAN sites, and 374.9 ± 481.2 mg/trap at high ALAN sites. Pairwise Tukey comparisons suggested all three ALAN levels were significantly different in insect biomass (medium—low estimate 0.442 ± 0.185, *p* = 0.044; high—low estimate 1.084 ± 0.167, *p* < 0.001; high—medium estimate 0.641 ± 0.145, *p* < 0.001). However, there was no insect biomass difference between open and cluttered sites (*p* = 0.135, Table 1), suggesting spatial complexity had no effect on insects. 

**Figure 2:**
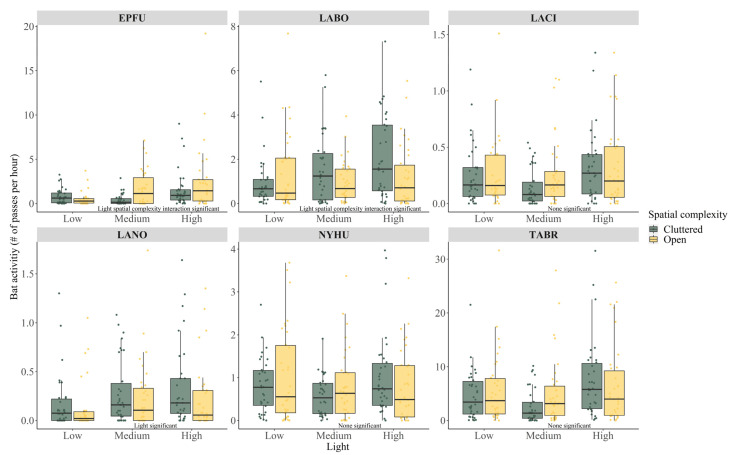
Boxplots (25th, 50th, 75th percentiles) and raw data point of species-specific bat activity at sites of varying ALAN levels and spatial complexity. Independent variable significance is annotated in the graph. Species abbreviations: big brown bat (*Eptesicus fuscus*, EPFU), eastern red bat (*Lasiurus borealis*, LABO), hoary bat (*Lasiurus cinereus*, LACI), silver-haired bat (*Lasionycteris noctivagans*, LANO), cave myotis (*Myotis velifer*, MYVE), evening bat (*Nycticeius humeralis*, NYHU), tricolored bat (*Perimyotis subflavus*, PESU) and Mexican free-tailed bat (*Tadarida brasiliensis*, TABR). Independent variable significance is annotated in the graph.

In 353,638 acoustic recording files, we were able to assign 119,154 call passes to 8 species (Table 2, Table S2). The species recorded most often was the Mexican free-tailed bat, followed by the eastern red bat, evening bat, big brown bat, hoary bat, and silver-haired bat. We further identified foraging calls for those species and conducted the GLM analysis for their activities and foraging ratios in relation to ALAN and spatial complexity. With regard to bat activity, not all species responded to ALAN or spatial complexity. For the hoary bat, the evening bat, and the Mexican free-tailed bat, species-specific bat activity did not differ among all sites, regardless of ALAN level or spatial complexity (all *p* > 0.05, Table 2). ALAN level affected silver-haired bat activity (Table 2, Figure 2). High ALAN sites with 0.3 ± 0.4 pass/hour was significantly higher than low ALAN site with 0.1 ± 0.3 pass/hour (Tukey high—low estimate 0.639 ± 0.270, *p* = 0.047, see Table S3 for other nonsignificant paired comparisons). There was no activity difference for silver-haired bats between cluttered and open sites (Table 2, Figure 2). 

Activity levels of both big brown bats and eastern red bats responded to ALAN, spatial complexity, and their interaction term (Table 2). Both species had higher activity as ALAN increased. However, their responses to spatial complexity were different. For the big brown bat, activity was not different between low ALAN cluttered and open sites, approximately 0.6 ± 0.8 pass/hour (Tukey open low –cluttered low estimate −0.419 ± 0.471, *p* = 0.946, Figure 2, Table S3). When ALAN increased to medium level, open sites had higher bat activity than cluttered sites (1.7 ± 2.0 pass/hour vs. 0.4 ± 0.6 pass/hour, Tukey open medium–cluttered medium estimate 1.363 ± 0.449, *p* = 0.027, Figure 2). Medium level ALAN open sites had the same amount of bat activity as high level ALAN sites (Tukey open medium –cluttered high estimate 0.026 ± 0.289, *p* = 1.000, Tukey open medium –open high estimate 0.404 ± 0.262, *p* = 0.621, Figure 2, Table S3). In contrast, an increase in eastern red bat activity in relation to ALAN was only found at cluttered sites (Tukey cluttered high–cluttered low estimate 0.749 ± 0.246, *p* = 0.028, Figure 2, Table S3). Cluttered high ALAN sites had the highest bat activity, approximately 2.1 ± 1.9 pass/hour. Open sites had the same amount of eastern red bat activity regardless of ALAN level (1.2 ± 1.4 pass/hour, all *p* > 0.05, Figure 2, Table S3).

**Figure 3:**
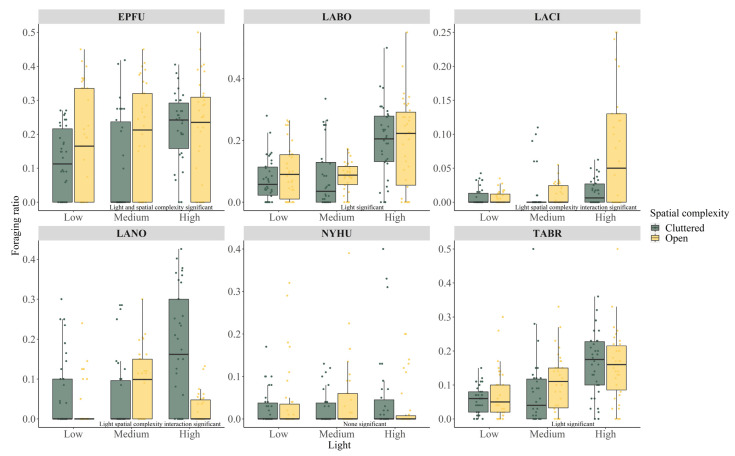
Boxplots (25th, 50th, 75th percentiles) and raw data points of species-specific foraging ratio at sites of varying ALAN levels and spatial complexity. Independent variable significance is annotated in the graph. Species abbreviations: big brown bat (*Eptesicus fuscus*, EPFU), eastern red bat (*Lasiurus borealis*, LABO), hoary bat (*Lasiurus cinereus*, LACI), silver-haired bat (*Lasionycteris noctivagans*, LANO), cave myotis (*Myotis velifer*, MYVE), evening bat (*Nycticeius humeralis*, NYHU), tricolored bat (*Perimyotis subflavus*, PESU), and Mexican free-tailed bat (*Tadarida brasiliensis*, TABR). Independent variable significance is annotated in the graph.

We identified a total of 12,178 foraging passes (Table 3). Proportionally, the big brown bat had the highest foraging ratio of 0.17 (Table S2), meaning that about 17% of identified big brown bat passes included foraging calls. The rank of overall foraging ratio for other species was eastern red bat (0.12), Mexican free-tailed bat (0.10), silver-haired bat (0.07), evening bat (0.04), and hoary bat (0.02). The GLM analysis for species-specific foraging ratio in relation to ALAN and spatial complexity again found that not all bats responded to ALAN or spatial complexity. The evening bat showed no difference in foraging ratio among all sites (All *p* > 0.05, Table 3, Figure 3). 

For the big brown bat, the eastern red bat, and the Mexican free-tailed bat, ALAN affected the foraging ratio independently from spatial complexity (all *p* < 0.05, Table 3, Figure 3). For the big brown bat, the foraging ratio was 0.21 ± 0.13 at high ALAN sites, significantly higher than low (0.15 ± 0.13) or medium (0.14 ± 0.16)_ALAN sites (Tukey high—low estimate 0.445 ± 0.174, *p* = 0.029, Tukey high–medium estimate 0.472 ± 0.175, *p* = 0.019, Figure 3, Table S3). A similar pattern was found in the eastern red bat and the Mexican free-tailed bat as well. Both species had significantly higher foraging ratio at high ALAN sites (eastern red bat foraging ratio high 0.19 ± 0.13, medium 0.08 ± 0.08, low 0.09 ± 0.08; Mexican free-tailed bat foraging ratio high 0.16 ± 0.10, medium 0.09 ± 0.10, low 0.06 ± 0.06; all Tukey comparison *p* < 0.05, Figure 3, Table S3). Furthermore, a higher foraging ratio was found for the big brown bat at open sites, independent of site ALAN level (open 0.19 ± 0.16 vs. cluttered 0.15 ± 0.13, Table 3, Figure 3), suggesting proportionally more foraging happened at open sites than at cluttered sites. 

The interaction between ALAN and spatial complexity was found to be significant for the hoary bat and the silver-haired bat (Table 3, Figure 3). For the hoary bat, only open sites with high ALAN levels had a significantly higher foraging ratio (0.07 ± 0.08) than all other sites (all foraging ratio < 0.02, all Tukey paired comparison *p* < 0.05, Figure 3, Table S3). For the silver-haired bat, even though foraging ratio generally increased with ALAN level (Table 3), the increase happened at cluttered high ALAN sites (cluttered low 0.06 ± 0.09, cluttered medium 0.08 ± 0.09, cluttered high 0.17 ± 0.15, Tukey cluttered high—cluttered low estimate 1.164 ± 0.302, *p* = 0.001, Tukey cluttered high—cluttered medium estimate 1.318 ± 0.317, *p* < 0.001, Figure 3, Table S3). In fact, the silver-haired bat foraging ratio at high ALAN open sites (0.02 ± 0.04) was no different from low ALAN cluttered sites (0.06 ± 0.09, Tukey open high—cluttered low estimate −0.990 ± 0.476, *p* = 0.286, Figure 3, see Table S3 for other paired comparisons). 

## 4. Discussion

Our results showed that ALAN affected insect biomass but not total insect count or Shannon diversity index. Consistent with many previous studies, an increase in insect biomass was positively correlated with the light intensity level of ALAN [26,69,70,71], potentially altering prey availability. A previous study showed that ALAN did not change the Shannon diversity index [72], which was also consistent with our result. Since different types of lights and different wavelengths of light affect different groups of insects [9,44,45,46], it was not surprising that the total number of insects might not be affected by ALAN. Very little is known about how urban spatial complexity might affect aerial insects, which led to our hypothesis that spatial complexity would have no effect on insects. Our results supported the original hypothesis. More studies on urban aerial insects are needed to understand what factors can affect their distribution. 

Predators’ responses to changes in prey availability can be an aggregation of individuals or increased foraging activity [73,74]. We recorded more acoustic activity of big brown bats, red bats, and silver-haired bats as the ALAN level increased. We also found that big brown bats, red bats, hoary bats, silver-haired bats, and Mexican free-tailed bats all foraged more often indicated by increased foraging ratios. No bat species in our study negatively responded to ALAN. Even though our results do not align with previous studies of ALAN and North American bats [24,36], they could be explained by the possibility that urban-dwelling bats might have adapted to ALAN. Considering that previous studies of North American bats were conducted in areas where ALAN was generally absent, urban bats in our studies were very likely to respond to ALAN differently, which might be another example of urban wildlife adapting to a city life [30,31,32]. Thus, our results were generally consistent with current knowledge of European bats and those bat species that might adapt to urban environments [33,75]. Our results for different levels of ALAN also indicate that light intensity should be considered when addressing ALAN impacts on wildlife [3,38,39,58]. Future studies should explore whether urban vs. non-urban individuals from the same species might respond to ALAN in different ways.

Beyond ALAN, our results showed that spatial complexity in urban environments played an important role in how bats foraged. Although the species in our study were likely open or edge foragers, their body sizes and wing morphologies were different. Therefore, their abilities to maneuver through cluttered space might differ, which might lead to the interaction between ALAN and spatial complexity [54,76]. For example, body mass has been found to be negatively correlated with clutter maneuvering ability [12,69]. In our study hoary bats and big brown bats have larger body sizes [55]. We found that the hoary bat foraging ratio increased only at open sites with high-level ALAN. Increases in big brown bat activity and foraging ratio were found only at open sites. In contrast, eastern red bats and silver-haired bats are relatively small [55]. Their positive responses to ALAN were mostly found at cluttered sites. This result demonstrated that predators would choose physically more suitable habitats to forage when prey availability was not a determining factor [77,78]. 

Interestingly, the interaction between ALAN and spatial complexity affected different species in different ways. For the big brown bat and the eastern red bat, the interaction term affected bat activity. For the hoary bat and the silver-haired bat, the interaction term affected the foraging ratio. We suspected that such a difference might indicate how different species adjusted foraging strategies differently when prey changed [73]. An increased acoustic activity might indicate more individuals present or the same individuals circling over the same spot more [41,79,80]. A higher foraging ratio on the other hand suggested more insect capture attempts being made. At open sites which were more suitable for the big brown bat and the hoary bat, two species responded to increased insect biomass in different ways for the same prey, each one adopting a different strategy. The same scenario was also found for the eastern red bat and the silver-haired bat at cluttered sites. Nevertheless, our results supported that the physical structure of the habitat could alter predator-prey relationships and exclude certain predators [74,77].

One aspect that our study was not able to address was how interspecific or intraspecific interactions among bats might affect species’ responses to ALAN. When optimal prey sources are available, bats might compete to dominate the prey source [81,82,83,84]. All species we examined were either open or edge foragers. There is no narrow-space forager species [57] such as Rafinesque’s big-eared bat (*Corynorhinus rafinesquii*) or fringed myotis (*Myotis thysanodes*) present in central Texas [67,70]. Species positively responding to cluttered habitats were edge foragers, which might be the result of competition as these species were less advantageous when competing with open foragers at open sites. Additionally, the evening bat was the only species in our study that showed no response to ALAN or spatial complexity. Considering this species is generally smaller than other species in the study area [55] and has been found less competitive with other species [81,82], we suspected that evening bat foraging activity might be opportunistic. Further study should address possible bat community dynamics under ALAN in relation to prey availability.

Recent research suggests that echolocating bats can cease producing commuting calls under different conditions, including avoiding sonar jamming or vision being available [5,85,86,87]. When and why bats might cease or reduce vocalizing are not completely known. Therefore, we could not rule out the possibility that reduced vocalizing might contribute to the changes in the foraging ratio at high ALAN sites. More studies that will employ both video and audio monitoring are needed to investigate this topic. Another topic that our study could not investigate was prey preference. Dietary preferences are known for many insectivorous bats [50,55,56]. How insects respond to ALAN is also group-specific [9,44]. Our insect sampling method might not capture the complete profile of prey. For example, we collected very few lepidopterans, which are known prey under ALAN [13,37,88]. Future studies should use more comprehensive prey sampling methods to better understand the prey availability and further investigate how prey preference might alter predator-prey relationships [89]. 

## 5. Conclusions

In contrast to our null hypothesis, we found that insect prey was positively correlated with ALAN but was not affected by urban spatial complexity. Five of six urban bat species responded to an increase in insect biomass, indicated by either elevated acoustic activity or foraging more often. However, their opportunity to use the prey resource was affected by spatial complexity. We found that large-sized big brown bats and hoary bats responded to change of prey in open sites whereas small-sized eastern red bats and silver-haired bats responded in cluttered sites. Our results suggest that the impact of ALAN on bats can vary when ALAN is coupled with urban spatial complexity and that foraging habitat suitability could alter which bat species might benefit from ALAN. We argue that urban bats might have adapted to cities and could be less prone to the negative impacts of light pollution. The absence of light-sensitive bat species in our study, however, should serve as evidence to limit and reduce light pollution when conserving light-sensitive species. Our study demonstrates the complexity of predator-prey relationships under multiple anthropogenic environmental changes in urban settings. We would like to highlight that general ecological principle still apply in novel urban ecosystems. Lastly, we suggest that future predator-prey relationship studies should consider prey preference and community-level predator competitions. 

## Figures and Tables

**Table 1:** General linear models results (regression estimate ± standard error on top, *p* value at bottom, *n* = 204) on insect variables in relation to ALAN, spatial complexity, and temperature.

Variable	Insect Count	Shannon Index	Insect Biomass
Total	18,822	N/A	45,776.5 (mg)
ALAN medium	−0.061 ± 0.106	0.184 ± 0.097	0.440 ± 0.185
	0.565	0.072	0.018
ALAN high	−0.000 ± 0.105	0.114 ± 0.009	1.084 ± 0.167
	0.996	0.160	<0.001
Open	0.005 ± 0.087	0.166 ± 0.065	0.188 ± 0.125
	0.953	0.115	0.135
ALAN medium × Open	N/A	N/A	N/A
ALAN high × Open	N/A	N/A	N/A
Temperature	0.039 ± 0.006	0.031 ± 0.004	0.068 ± 0.009
	<0.001	<0.001	<0.001

**Table 2:** General linear models results (regression estimate ± standard error on top, *p* value at bottom, *n* = 204) on species-specific bat activity in relation to ALAN, spatial complexity, and temperature.

Variable	EPFU	LABO	LACI	LANO	NYHU	TABR
Total passes identified	11,608	18,223	3802	2473	12,229	70,318
ALAN medium	−0.598 ± 0.499	0.509 ± 0.258	−0.266 ± 0.199	0.562 ± 0.274	−0.274 ± 0.163	−0.296 ±0.209
	0.232	0.047	0.183	0.042	0.095	0.159
ALAN high	0.739 ± 0.361	0.749 ± 0.246	0.172 ± 0.178	0.640 ± 0.270	0.025 ± 0.151	0.284 ± 0.181
	0.042	0.003	0.334	0.019	0.865	0.119
Open	−0.419 ± 0.478	0.375 ± 0265	0.182 ± 0.153	−0.335 ± 0.205	0.159 ± 0.130	0.162 ± 0.156
	0.376	0.158	0.235	0.105	0.222	0.302
ALAN medium × Open	1.781 ± 0.651	−0.819 ± 0.370	N/A	N/A	N/A	N/A
	0.007	0.028				
ALAN high × Open	0.849 ± 0.541	−0.771 ± 0.352	N/A	N/A	N/A	N/A
	0.118	0.030				
Temperature	0.009 ± 0.014	0.052 ± 0.010	−0.006 ± 0.010	−0.013 ± 0.014	0.043 ± 0.009	0.005 ± 0.010
	0.521	<0.001	0.541	0.352	<0.001	0.610

**Table 3:** General linear models results (regression estimate ± standard error on top, *p* value at bottom, *n* = 204) on species-specific foraging ratio in relation to ALAN, spatial complexity, and temperature.

Variable	EPFU	LABO	LACI	LANO	NYHU	TABR
Total foraing passes	1920	2212	81	175	438	7352
ALAN medium	−0.028 ± 0.187	−0.037 ± 0.187	0.551 ±0.520	−0.154 ±0.373	−0.309 ± 0.377	0.382 ± 0.184
	0.880	0.845	0.290	0.680	0.413	0.039
ALAN high	0.445 ± 0.174	0.951 ± 0.160	0.701 ± 0.507	1.163 ± 0.302	0.063 ± 0.345	1.014 ± 0.168
	0.012	<0.001	0.168	<0.001	0.855	<0.001
Open	0.296 ± 0.145	0.066 ± 0.131	−0.149 ± 0.607	−0.750 ± 0.440	0.263 ± 0.297	0.098 ± 0.131
	0.043	0.617	0.807	0.099	0.377	0.457
ALAN medium × Open	N/A	N/A	0.007 ± 0.763	1.263 ± 0.562	N/A	N/A
			0.993	0.026		
ALAN high × Open	N/A	N/A	1.817 ± 0.687	−1.403 ± 0.618	N/A	N/A
			0.009	0.024		
Temperature	0.000 ± 0.009	−0.007 ± 0.010	−0.018 ± 0.013	0.009 ± 0.013	0.079 ± 0.022	−0.025 ± 0.009
	0.981	0.424	0.179	0.519	<0.001	0.003

## Data Availability

The data presented in this study are openly available in FigShare at 10.6084/m9.figshare.19287614.

## Supplementary Materials

The following supporting information can be downloaded at: https://www.mdpi.com/article/10.3390/biology11060829/s1, File S1: photo illustration of study sites; Table S1: summary table of insects collected in the study; Table S2: summary table of bats detected in the study; Table S3: Raw R outputs of Tukey post hoc comparisons and Mantel’s test.
