# Supplementary material for: Predator-Prey Relationship between Urban Bats and Insects Impacted by Both Artificial Light at Night and Spatial Clutter
Source: Biology (Basel). 2022 May 27;11(6):829. doi: 10.3390/biology11060829 (PMC9219930; doi:10.3390/biology11060829)

Supplementary Materials File S1: photo illustration of study sites for Li and Wilkins

The two photos (page 1 and 2) below demonstrate cluttered vs. open sites.

Clutter site: tall fence, about 5-6 meters. The fence separates a sport field into parts. Additionally, this is a high ALAN site because of lights for illumination.

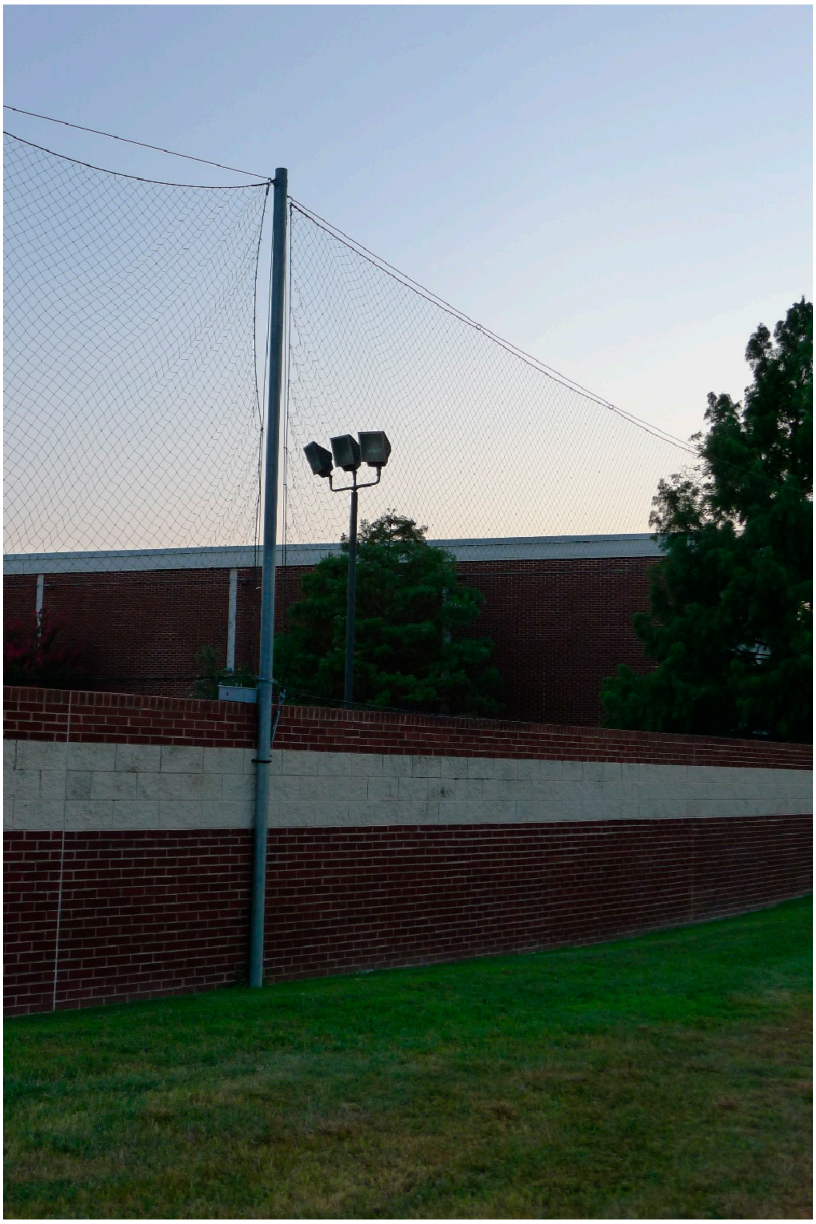

Open site: parking facility top level besides light poles, no object taller than 3m. This is also a high ALAN site due to all the lights.

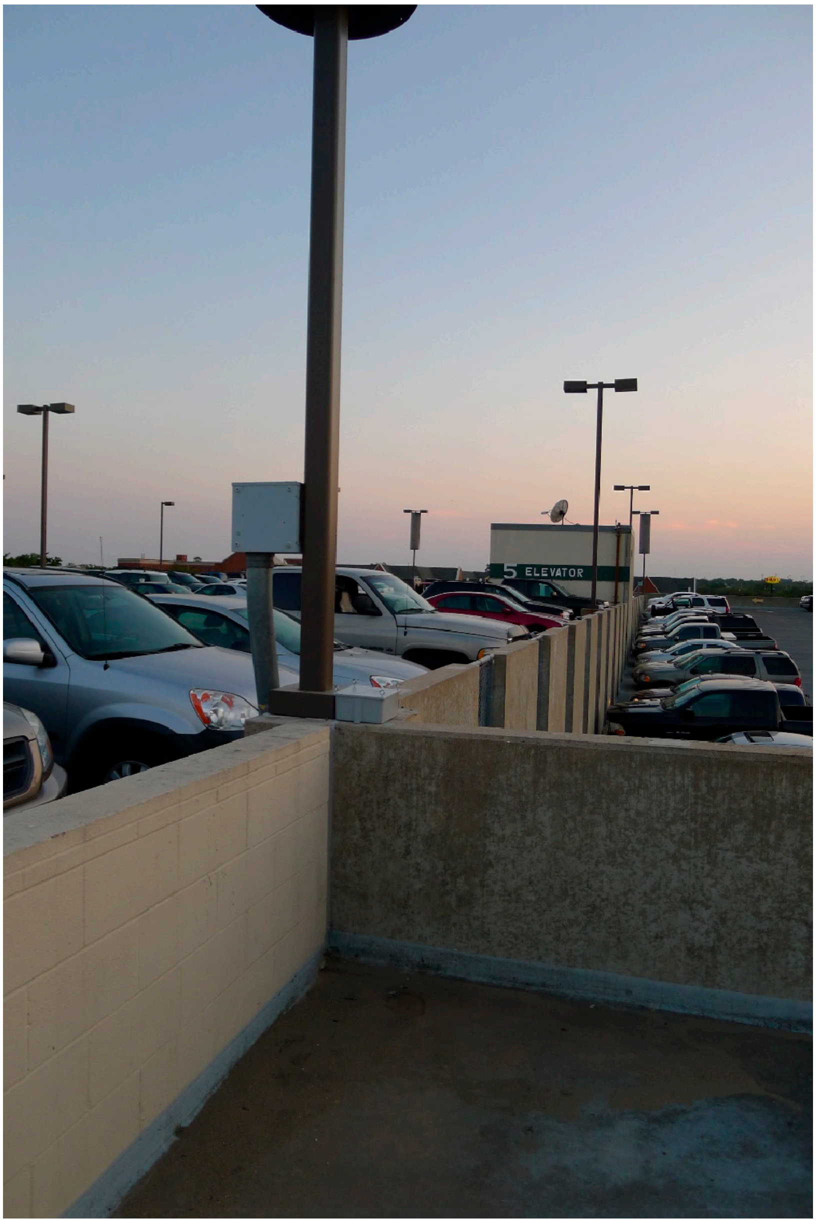

The satellite image below shows a low ALAN open site:

The facility is about 100 meters from the site. The pin (yellow) is the location for detector. It is about 60 meters away from the powerline or the nearest tree.

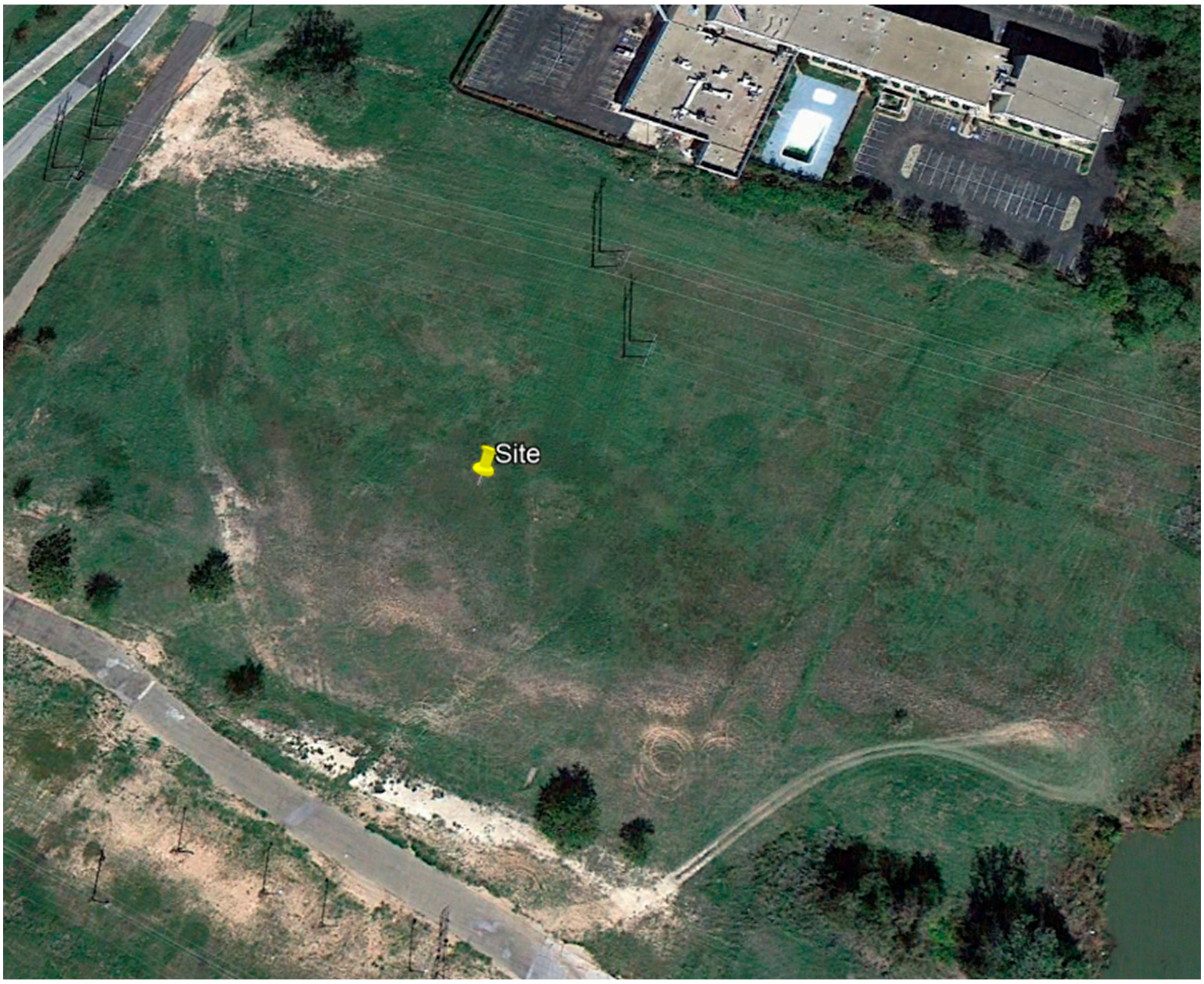

The satellite image below shows a low ALAN cluttered site:

Isolated buildings and stacked construction materials form the clutter in the space.

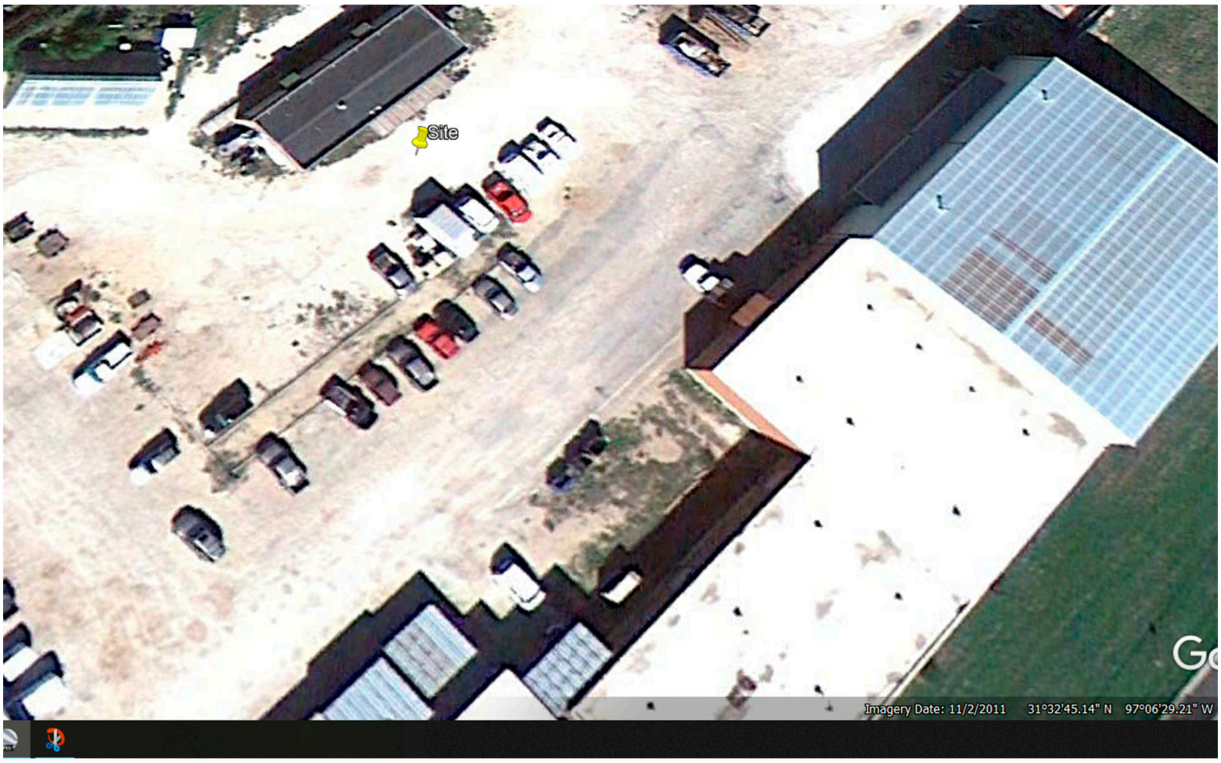

The satellite image below shows a medium ALAN cluttered site:

The clutter is formed by scattered trees

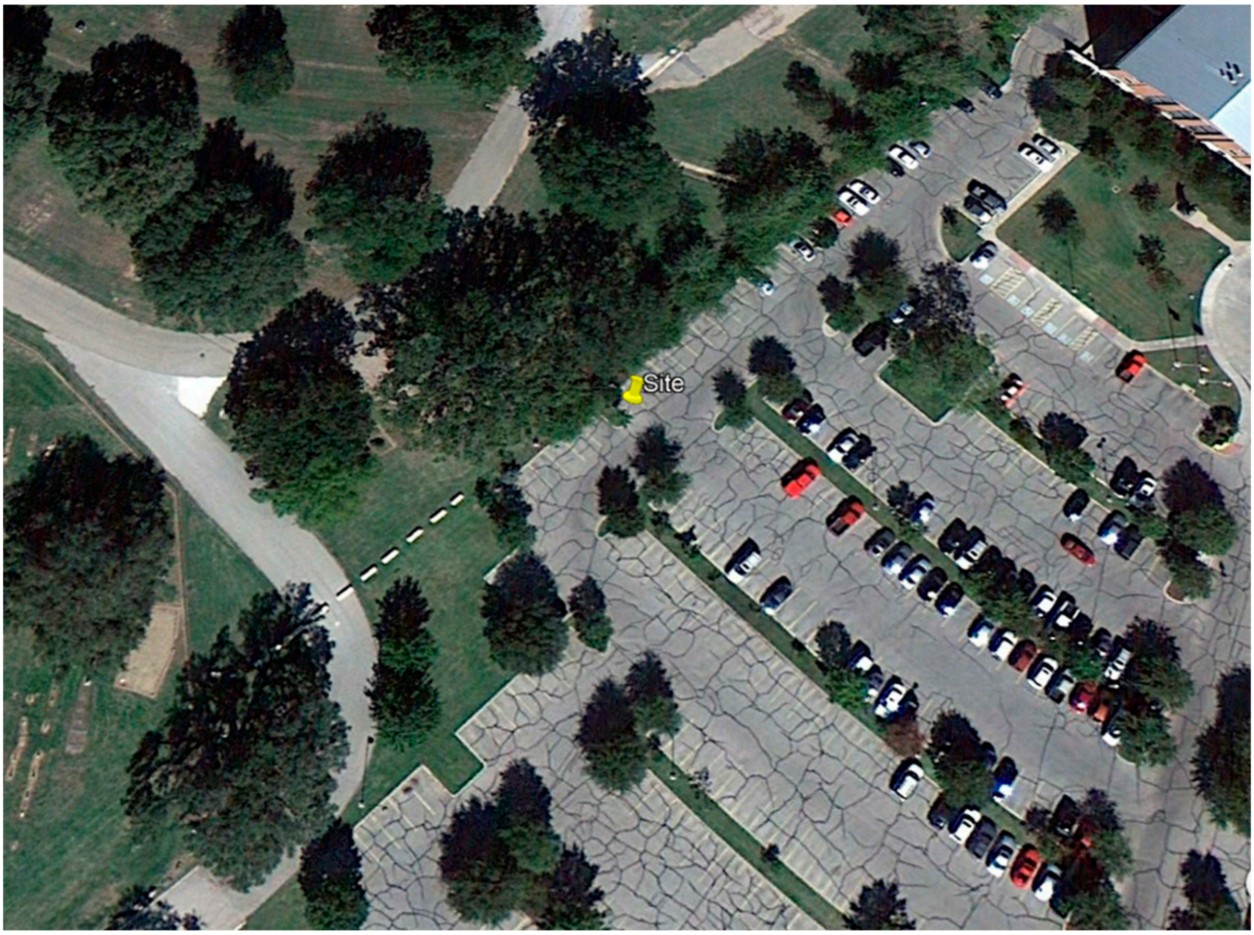

The satellite image below shows a medium ALAN open site:

This site is on top of a park deck. The location of the detector is far away from the building edge. This is a relatively new parking structure and the light was somewhat dimmed as compared to other parking structures in the area.

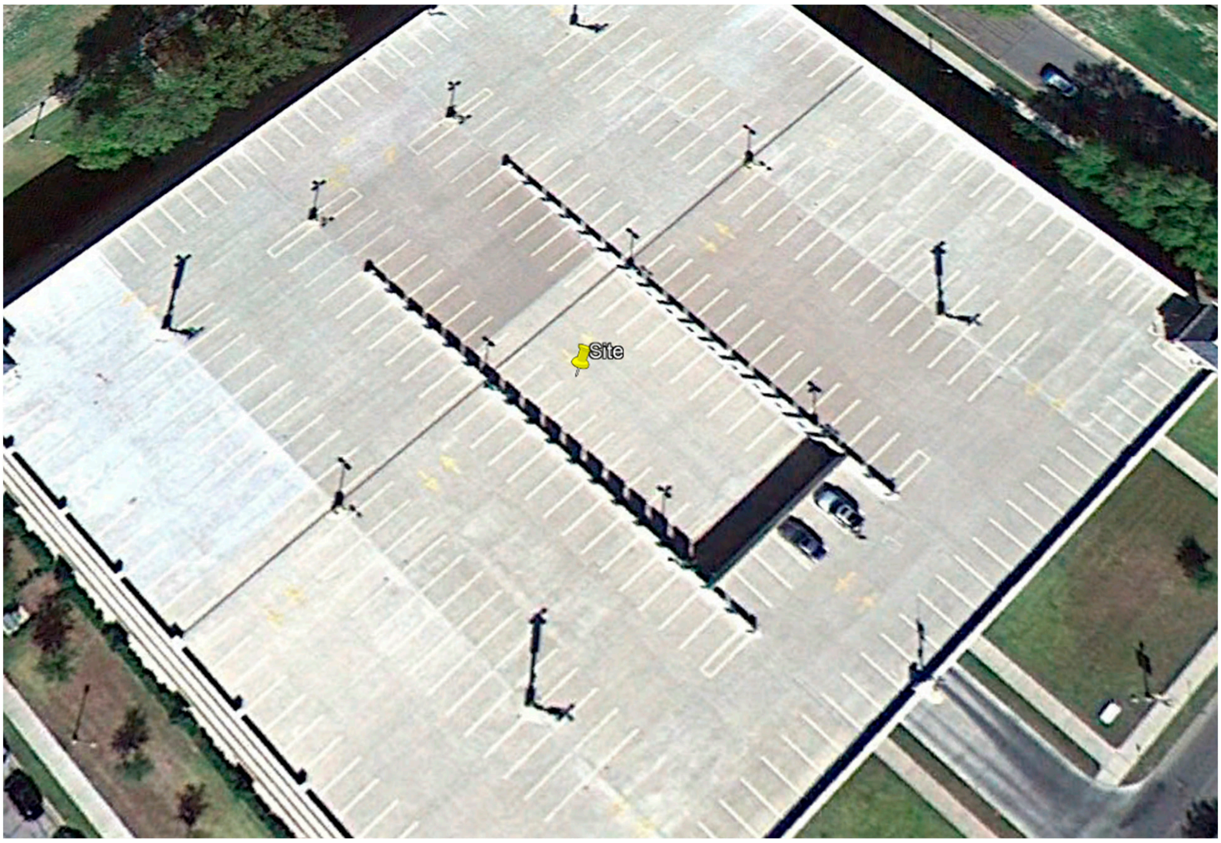

The satellite image below shows a high ALAN cluttered site:

Trees, fences, sculptures form a cluttered space.

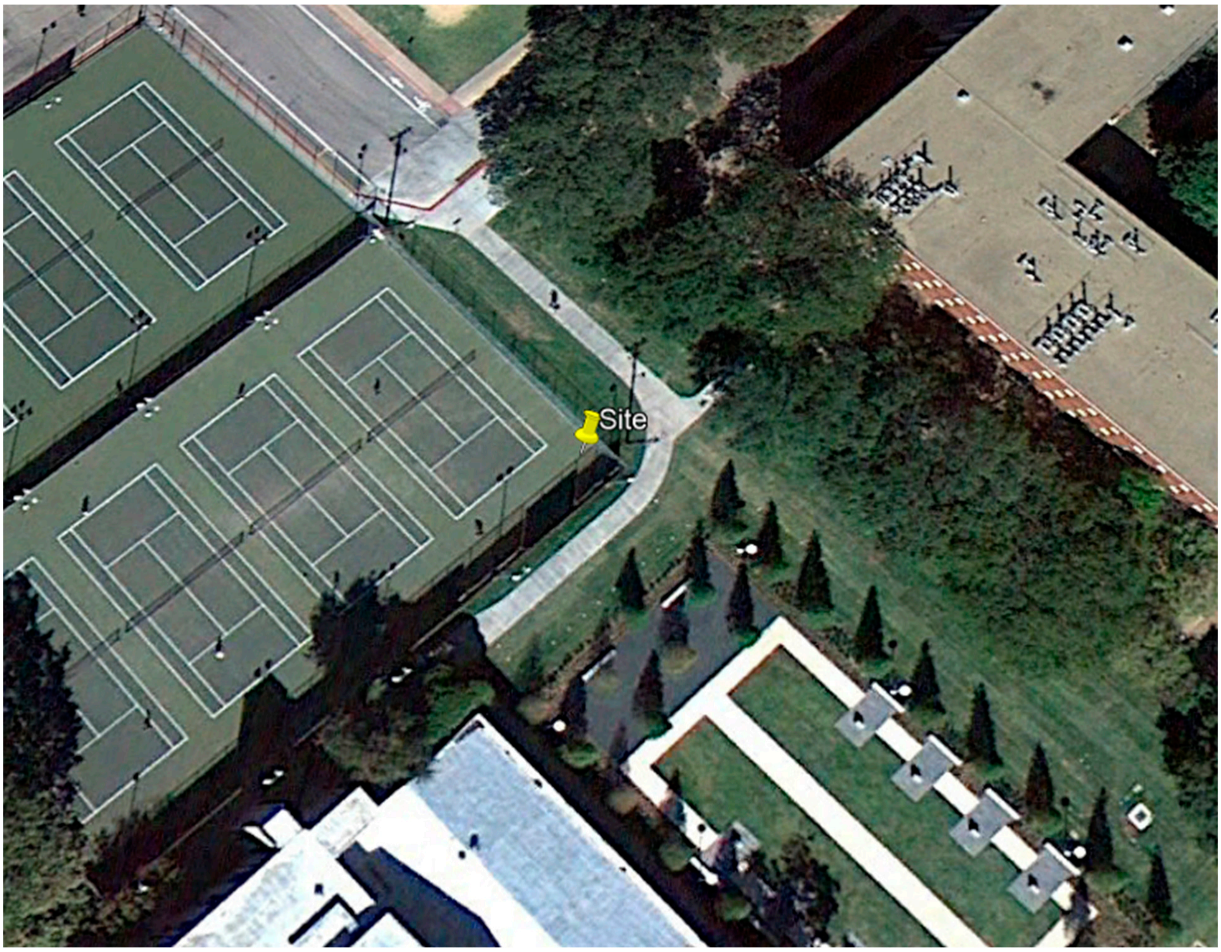

The satellite image below shows a high ALAN open site:

High ALAN comes from the parking lot lights and the nearby stadium.

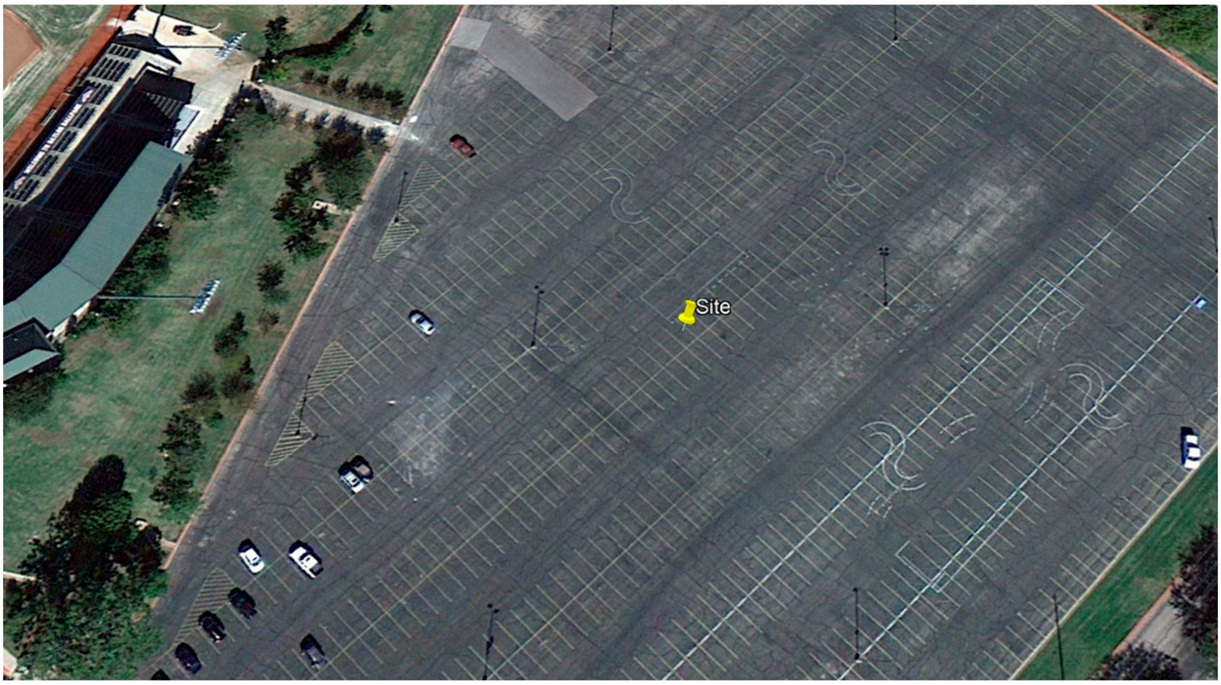

Supplement: Supplementary file 1 [file biology-11-00829-s001.zip › File S1.pdf]
