# Supplementary material for: Predator-Prey Relationship between Urban Bats and Insects Impacted by Both Artificial Light at Night and Spatial Clutter
Source: Biology (Basel). 2022 May 27;11(6):829. doi: 10.3390/biology11060829 (PMC9219930; doi:10.3390/biology11060829)
Supplement: Supplementary file 1 [file biology-11-00829-s001.zip › Table S1.pdf]

Table S1. Number and order of aerial insects captured on glue traps, and their total biomass and mean individual biomass recorded between September 2011 and October 2012 on Baylor University campus, Waco, McLennan Co., Texas, USA. Only insects with body length 3 – 30 mm were identified to order. Small insects included all insects with body length 1 – 3 mm.

Order	Total count	Total biomass (mg)	Total biomass percentage	Mean biomass (mg / organism)
Coleoptera	634	17173.7	37.5%	27.09
Diptera	3580	10080.6	22.0%	2.82
Ephemeroptera	53	2014.4	4.4%	38.01
Hemiptera	61	4328.3	9.5%	70.96
Homoptera	277	1801.3	3.9%	6.50
Hymenoptera	379	1637.4	3.6%	4.32
Lepidoptera	82	2246.8	4.9%	27.40
Neuroptera	63	1085.1	2.4%	17.22
Odonata	14	1153.8	2.5%	82.41
Plecoptera	78	1191.1	2.6%	15.27
Trichoptera	61	525.3	1.1%	8.61
Small insects	13540	2538.7	5.5%	0.19
Total	18822	45776.5	100.0%	N/A
