# Supplementary material for: Predator-Prey Relationship between Urban Bats and Insects Impacted by Both Artificial Light at Night and Spatial Clutter
Source: Biology (Basel). 2022 May 27;11(6):829. doi: 10.3390/biology11060829 (PMC9219930; doi:10.3390/biology11060829)
Supplement: Supplementary file 1 [file biology-11-00829-s001.zip › Table S2.pdf]

Table S2. Number of bat passes, foraging passes, and overall foraging ratio recorded between September 2011 and October 2012 on Baylor University campus, Waco, McLennan Co., Texas, USA.

Bat species	Total passes	Foraging passes	Foraging ratio
Eptesicus fuscus	11608	1920	0.17
Lasiurus borealis	18223	2212	0.12
Lasiurus cinereus	3802	81	0.02
Lasionycteris noctivagans	2473	175	0.07
Myotis velifer	202	N/A	N/A
Nycticeius humeralis	12229	438	0.04
Perimyotis subflavus	299	N/A	N/A
Tadarida brasiliensis	70318	7352	0.10
